# Two Nearly Complete Nosocomial Pathogen Genome Sequences Reconstructed from Early-Middle 20th-Century Dental Calculus

**DOI:** 10.1128/MRA.00850-20

**Published:** 2020-10-22

**Authors:** LaShanda Williams, Carla Cugini, Siobain Duffy

**Affiliations:** a Department of Ecology, Evolution, and Natural Resources, Rutgers University, New Brunswick, New Jersey, USA; b Department of Oral Biology, Rutgers School of Dental Medicine, Newark, New Jersey, USA; University of Arizona

## Abstract

Acinetobacter baumannii and Stenotrophomonas maltophilia genomes were reconstructed from early-middle 20th-century human skeletal remains, maintained in natural history museums, using a metagenomic binning approach.

## ANNOUNCEMENT

Evolutionary and phylogenetic analyses of historical and modern strains of nosocomial pathogens can provide insight into the acquisition and origin of antibiotic resistance genes. Nosocomial infections present a major health burden worldwide, accounting for 7% and 10% of infectious diseases in developing and developed nations, respectively (1). Additionally, many Gram-negative nosocomial pathogens, such as Acinetobacter baumannii and Stenotrophomonas maltophilia, are resistant to many antibiotics, making successful treatment difficult (2). Here, we announce the publication of two mostly complete (estimated >95%) metagenome-associated genomes (MAGs) of A. baumannii and S. maltophilia.

Dental calculus is formed from periodic mineralization of plaque on dentition (3, 4). It is composed of the microbial constituents of dental plaque in addition to salivary calcium phosphate ions, gingival crevicular fluid, host biomolecules (5), and dietary biomolecules (3, 4, 6 – 8). It is formed in life and remains present after death, and it has been used to assess ancient and historical mammalian oral microbiota (8 – 14). We obtained dental calculus from skeletal remains of an individual who died at the beginning of the 20th century, housed at the National Museum of Natural History (NMNH) (sample number 364459), and one who died in the middle of the 20th century, housed at the American Museum of Natural History (AMNH) (sample number AMNH98137_Q01). On a sanitized table, dental calculus was scraped from posterior teeth using a sterilized dental scaler and toothbrush and was placed in a 2-ml microcentrifuge tube. Samples were then brought to the Laboratories of Molecular Anthropology and Microbiome Research at the University of Oklahoma for molecular analysis. The surface of the calculus sample was decontaminated with UV light prior to incubation for 48 h in 1 ml of 0.5 M EDTA solution with 100 µl of Qiagen proteinase K. DNA was purified with the Qiagen PCR purification MinElute kit, and shotgun libraries were constructed using the NEBNext kit. Sequencing was completed on an Illumina HiSeq system with a 2 × 150-bp configuration at the Yale Center for Genome Analysis.

Sequencing quality for each sample was checked with FastQC (v.0.11.8) (15), adapters were removed using AdapterRemoval (v.2.1.7) (16), and human-associated DNA was removed from samples using KneadData (March 2019 release) (https://huttenhower.sph.harvard.edu/kneaddata). Following the removal of human-associated reads, we used MEGAHIT (v.1.0) (17) to assemble reads into contigs, which were then uploaded into PATRIC (v.3.5.31) (https://www.patricbrc.org) for metagenomic binning analysis (binning metrics determined using EvalG are presented in Table 1) (18 – 21). PATRIC uses a binning algorithm that scans contigs for single-copy marker genes and then calculates a gene marker similarity metric to recruit similar genomes present in the PATRIC reference database (18, 19, 21). Each sample was run independently, and PATRIC revealed significant numbers of reads in sample AMNH98137_Q01 that were likely A. baumannii, as well as S. maltophilia in sample 364459. mapDamage (v.2.0) (22) was used to authenticate the historical origins of the assemblies, and patterns characteristic of DNA damage at the sequence ends were found when the assemblies were mapped against reference genomes (A. baumannii assembly number ASM74664v1 and S. maltophilia assembly number ASM7248v1). Mapping to the reference genomes provided a scaffold for the relevant contigs to be compiled into a MAG. Annotation was performed by the NCBI using the Prokaryotic Genome Annotation Pipeline (PGAP) (v.4.2, best-placed reference protein set) (23). Default parameters were used for all software unless otherwise specified.

**TABLE 1:** Sequence statistics and metagenomic binning statistics for each bacterial genome

Parameter	Data for:	
	Acinetobacter baumannii	Stenotrophomonas maltophilia
Calculus sample no.	AMNH98137_Q01	364459
Museum (city)	AMNH (New York)	NMNH (Washington, DC)
Museum collection	Medical collection	Kodiak Island collection
Total no. of reads generated	1,605,869	7,410,027
Avg read length (bp)	83.22	107.20
Total no. of contigs mapped to pathogen reference genome	1,101	9,976
Total length of reconstructed sequence (bp)	3,480,250	4,538,581
Completeness, relative to reference sequence (%)	95.3	98.1
Coarse consistency (%)	98.2	97.7
Fine consistency (%)	92.8	91.4
Contamination (%)	7.2	9.3
Mean coverage (fold)	7.84	16.94
GC content (%)	39.2	66.35
No. of coding regions	3,832	4,815

Of note, the AdaBoost classifier within PATRIC characterized the A. baumannii assembly as showing a carbapenem resistance phenotype due to the presence of a resistance gene (accuracy, 0.936; F1 score, 0.942; area under the curve [AUC], 0.957). Carbapenem was not introduced until 1983, and all modern strains carry this gene; this reconstruction supports the idea that the carbapenem resistance phenotype arose prior to the antibiotic revolution (24).

### Data availability.

These A. baumannii and S. maltophilia genome sequences have been deposited in GenBank under the accession numbers JACBON000000000 and JACBOO000000000, respectively. The versions described here are the first versions. All metagenomic data generated from this announcement are available under BioProject number PRJNA643812. Raw whole-genome shotgun data generated from each sample were deposited in the SRA under the accession numbers SRR12136712 and SRR12136713. Metagenomic bins can be found under BioSample numbers SAMN15430098 and SAMN15430099.
